# Green space and schizophrenia: A review

**DOI:** 10.1192/j.eurpsy.2021.1414

**Published:** 2021-08-13

**Authors:** R. Mota Freitas, M.T. Valadas

**Affiliations:** 1 Departamento De Psiquiatria E Saúde Mental, Hospital do Espírito Santo de Évora, Évora, Portugal; 2 Serviço De Psiquiatria, Unidade Local de Saúde do Baixo Alentejo, Beja, Portugal

**Keywords:** schizophrénia, Green space

## Abstract

**Introduction:**

Urban living has consistently been associated with higher risk of developing schizophrenia when compared to rural living. Exposure to green space has been associated with better mental health outcomes and, more recently, childhood exposure to green space has been linked with lower rates of schizophrenia. The reasons for these findings remain unknown, although lower levels of pollution and psychological factors may play a role.

**Objectives:**

We aim to review the literature regarding exposure to green space and its relationship with the risk of developing schizophrenia.

**Methods:**

We performed an updated review in the PubMed database using the terms “green space” and “schizophrenia”. The included articles were selected by title and abstract.

**Results:**

Growing up surrounded by non-urban environments is associated with lower schizophrenia rates. Upbringing in urban areas is associated with higher schizophrenia rates when compared with non-built-up areas. Schizophrenia risk seems to decrease with vegetation density in a dose-response relationship for urban and agricultural areas. Risk of schizophrenia has been found to be associated additively with green space exposure and genetic liability. No evidence for gene-environment interaction has been reported so far in this regard.

**Conclusions:**

Exposure to green space during childhood appears to lower the risk of developing schizophrenia later in life and can be a preventive strategy. Further research in this area is needed.

